# Longitudinal Intergenerational Birth Cohort Designs: A Systematic Review of Australian and New Zealand Studies

**DOI:** 10.1371/journal.pone.0150491

**Published:** 2016-03-18

**Authors:** Michelle L. Townsend, Angelique Riepsamen, Christos Georgiou, Victoria M. Flood, Peter Caputi, Ian M. Wright, Warren S. Davis, Alison Jones, Theresa A. Larkin, Moira J. Williamson, Brin F. S. Grenyer

**Affiliations:** 1 Illawarra Health and Medical Research Institute, University of Wollongong, Wollongong, NSW, Australia; 2 School of Psychology, University of Wollongong, Wollongong, NSW, Australia; 3 School of Women's and Children's Health, Discipline of Obstetrics and Gynaecology, University of New South Wales, Sydney, NSW, Australia; 4 Eastern Health, Melbourne, Victoria, Australia; 5 Monash University, Faculty of Medicine, Nursing and Health Services, Eastern Health Clinical School, Melbourne, Victoria, Australia; 6 Graduate School of Medicine, University of Wollongong, Wollongong, NSW, Australia; 7 Faculty of Health Sciences, University of Sydney, Sydney, NSW, Australia; 8 St Vincent’s Hospital, Darlinghurst, NSW, Australia; 9 Illawarra Shoalhaven Local Health District, NSW Health, Sydney, NSW, Australia; 10 School of Nursing and Midwifery, Central Queensland University, Rockhampton, Queensland, Australia; Seattle Childrens Hospital, UNITED STATES

## Abstract

**Background:**

The longitudinal birth cohort design has yielded a substantial contribution to knowledge of child health and development. The last full review in New Zealand and Australia in 2004 identified 13 studies. Since then, birth cohort designs continue to be an important tool in understanding how intrauterine, infant and childhood development affect long-term health and well-being. This updated review in a defined geographical area was conducted to better understand the factors associated with successful quality and productivity, and greater scientific and policy contribution and scope.

**Methods:**

We adopted the preferred reporting items for systematic reviews and meta-analyses (PRISMA) approach, searching PubMed, Scopus, Cinahl, Medline, Science Direct and ProQuest between 1963 and 2013. Experts were consulted regarding further studies. Five inclusion criteria were used: (1) have longitudinally tracked a birth cohort, (2) have collected data on the child and at least one parent or caregiver (3) be based in Australia or New Zealand, (4) be empirical in design, and (5) have been published in English.

**Results:**

10665 records were initially retrieved from which 23 birth cohort studies met the selection criteria. Together these studies recruited 91,196 participants, with 38,600 mothers, 14,206 fathers and 38,390 live births. Seventeen studies were located in Australia and six in New Zealand. Research questions initially focused on the perinatal period, but as studies matured, longer-term effects and outcomes were examined.

**Conclusions:**

This review demonstrates the significant yield from this effort both in terms of scientific discovery and social policy impact. Further opportunities have been recognised with cross-study collaboration and pooling of data between established and newer studies and international studies to investigate global health determinants.

## Introduction

The scientific yield from longitudinal birth cohort studies is impressive, particularly as such designs have found that genetic factors and environmental exposures prenatally and in early life can have long–term consequences for not only adult physical health but also mental health and well-being [1–8]. A significant international focus on the ‘Developmental Origins of Health and Disease’[9] (DOHaD) is developing; exposures peri-conception and in-utero have been linked with adult diseases including stroke, type 2 diabetes [10], cardiovascular disease [11] and other metabolic disorders [12]. Exposure to stress and adversity is also acknowledged as a negative and pervasive influence on the developing fetus and child [13–16]. For example, abuse and chronic stress exposure in utero, infancy and childhood, have been linked to poorer physical and mental adaptation throughout the life-span [13, 17, 18] as well as poorer socioeconomic, legal and educational outcomes.

Despite the increasing appreciation of the importance of understanding and addressing the lifelong legacy of the peri-conceptional, in-utero and early childhood periods across the human life course, there remain many gaps in the scientific understanding of mechanisms influencing these formative phases. The transgenerational susceptibility to health problems is now emerging as a focus of research. Silveira et al. [19] argue for the integration of genetic, epigenetic and environmental variables in future studies. Internationally several large and high quality birth cohort designs have created biobank repositories of biological specimens to support the investigation of the interplay between genetic, lifestyle and environmental factors in health and well-being [20]. As well as the role of genetics in transgenerational research, epigenetic changes are emerging as a key component. These heritable chemical modifications to the DNA structure regulate gene expression independently of the DNA sequence, and are susceptible to modifications by environmental factors such as diet, lifestyle, chemical exposure, intrapartum period and behavioural interactions (e.g. maternal attachment) [21, 22].

This review focuses on Australian and New Zealand birth cohort studies. The studies undertaken in these developed regions are characterised by particular social ecologies and policy environments that differ from other countries, with the populations eligible for universal and quality health care and education, living in clean environments with less defined class based society and diverse cultural backgrounds.

This current systematic review aims to review emerging and existing birth cohort studies in Australia and New Zealand and is informed by the review undertaken by Nicholson and Rempel in 2004 [23]. Like Nicholson and Rempel's work, we aim to understand factors associated with successful designs, including those that yield increased quality and productivity, and greater scientific and policy contribution and scope. The previous 2004 review identified 13 birth cohorts. The review was used to influence the development and implementation of the Longitudinal Study of Australia’s Children (LSAC) [24]. In the decade since the Nicolson and Rempel article, efforts have been made to increase collaboration between the various longitudinal studies, particularly in Australia through the Australian Research Alliance for Children and Youth (ARACY) longitudinal studies network, and other groups such as the ACAORN: Australasian Child and Adolescent Obesity Research Network. However there has been little published work overviewing the current research effort, not only in terms of recent and emerging studies, but also with respect to collaborative efforts that use pooled data sets.

The objectives of this article are to systematically review Australian and New Zealand birth cohort studies to understand the characteristics and yield of the established and emerging studies, and to inform the development of future research.

## Methods

This review followed the Preferred Reporting Items for Systematic Reviews and Meta-Analysis (PRISMA) Statement [25] and Sutton et al. [26] guidelines for conducting and reporting systematic reviews. Methods of data collection and inclusion criteria were predetermined.

### Data Sources

Studies were identified in two phases: (i) searching electronic databases, (ii) consultation with experts in the area of research. These were both completed from March to October 2013.

Searching electronic databases: PubMed, Scopus, Cinahl, Medline, Science Direct and ProQuest databases were searched for eligible records. Search terms used for each database included “(birth cohort or longitudinal) and (prenatal, antenatal, infan*, birth* or pregnan*), limited by Austral* or New Zealand.”

Consultation with experts: A list of the identified cohort studies was sent to experts in the area of research inviting contribution of any further studies that may meet criteria. Experts were determined as having authored or co-authored ten or more journal articles in the field relevant to the study criteria (e.g. as first or senior author). Where multiple experts were identified within the same study, institutional websites were appraised to determine the most suitable (active, senior) author to contact. When an automatically generated out of office email response was received a second expert from the study was identified.

### Study Selection

**Fig 1:**
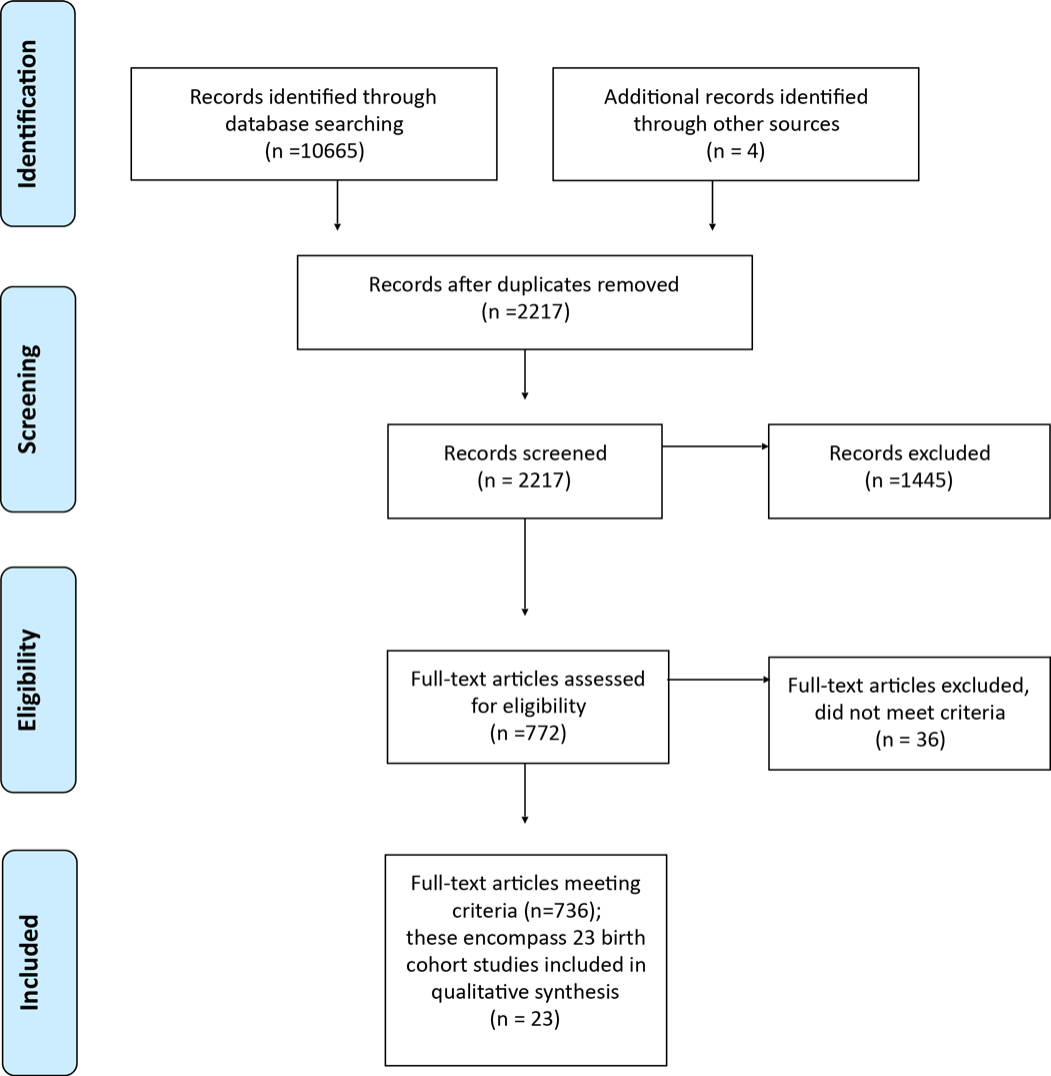
Flow diagram of information through the different phases of the systematic review.

One author (MT) independently carried out the identification of records and data extraction over a two month period, which was then independently reviewed by a second author (AR). The second author was blind to prestige factors including authors, institutions, journal titles and publisher details. The search strategies, eligibility criteria and data extraction forms were piloted before use. The two authors compared their findings and discrepancies were discussed and resolved to reach a consensus. A list of the data that was extracted is shown in Fig 1. Data related to risk of bias was collected to help explain any discrepancy in results and to assess the strength of evidence. The inclusion criteria were as follows: (1) Data was obtained on the child and at least one parent or caregiver (usually mother and baby), (2) Cohort recruited from the general population in Australia or New Zealand, (3) Cohort recruited in the perinatal period (up to 6 weeks post-partum), (4) Empirical studies (excluding publications that were anecdotes, reviews, book chapters, letters to the editor and editorials); and (5) Publication was in English. The exclusion criteria were as follows: (1) There were no consent procedure or the study was based solely on medical records, (2) Cohort focused on a single disease phenotype or clinical sample.

Inclusion required the identified study to meet all five criteria. The aim of this systematic review was to identify all birth cohort studies, not necessarily all publications related to each study.

### Data Extraction and Analysis

A data collection tool was developed to document and then later aggregate information about specific variables of interest including (i) the study details, and (ii) the study methodology. One author (MT) extracted data from the included studies by reviewing published papers, reports, book chapters, websites and personal correspondence. Extraction of data regarding the study details included details of the study source, commencement, design, aims, number of participants, data collection waves, status of the study, institution, location of the study and retention rates. Data extraction regarding the study methodology examined the methods of the study, types of measures used and which members of the study participated in the various research activities. This analysis was undertaken through a combination of review of individual articles and correspondence with the study authors. For each study the authors identified a protocol paper, where available, and the first and most recent publications were located and reviewed to inform the data analysis. Further articles were reviewed as required. Quantitative data was analysed in SPSS using descriptive statistics. Qualitative data was analysed using NVivo Version 10.

## Results

Search of electronic databases: We identified 10665 articles from six databases: Pubmed (3881), Scopus (3201), Medline (2976), Science Direct (339), Cinahl (235) and Proquest (33). Of these, 8452 were duplicates and so 2213 publications were screened (Fig 1). Of these, 1445 were excluded after inspection of their title, abstract or full-text as not meeting the inclusion criteria. 22 studies were identified from the remaining 772 articles as meeting inclusion criteria and were included in the systematic review.

Consultation with experts: Nineteen experts were identified and contacted, with nine responses. This phase resulted in four further studies being identified. Three of these failed to meet the inclusion criteria but one further birth cohort study (the Babies, Bumps and Beyond study) was identified and included, thus a total of 23 studies were included in the systematic review (Fig 1). The characteristics of the studies are provided in Table 1. Two very large cohort studies were excluded as they did not meet the intergenerational criteria but further information on these studies can be obtained from [27–29] and [30].

**Table 1:** Characteristics of Included Studies Grouped by Recruitment Age.

Study name	Study design	Demographics	Recruitment	Sample size	Aims	Data Collection	Source
Environments for Health Living Griffith Birth Cohort Study	Repeated sample prospective longitudinal birth cohort	*Location*: South East Queensland, Northern New South Wales, Australia. *Year & status*: 2006; Ongoing	*Period*: 4 months of the year over 5 years. *Age of*: Antenatal	*Initial total*: 2879 *Mothers*: 2879 *Babies*: 2904 *Partners*: 2879	To study the social and environmental factors, neighbourhood and family functioning, maternal lifestyle and in utero exposures on child health and development outcomes.	*Waves & Ages*: 5; Antenatal, birth, 1,3,5 y. *Retention rate*: N/A	[31, 32]
Generation 1 Cohort	Prospective, longitudinal birth cohort	*Location*: Adelaide, South Australia, Australia. *Year & status*: 1998; Ongoing	*Period*: 24 months. *Age of*: Antenatal	*Initial total*:557 *Mothers*: 557 *Babies*: 557 *Partners*: Not stated	To study early life influences on obesity and fat patterning in children: critical periods, environmental determinants, and socio-cultural context.	*Waves & Ages*: 8; Antenatal, 6,9,12 m, 2,3.5,5.5 y *Retention rate*: 72% at 9 y	[33–35]
Growing up in New Zealand	Prospective, longitudinal birth cohort	*Location*: Auckland, North Island, New Zealand. *Year & status*: 2009; Ongoing	*Period*: 12 months. *Age of*: Antenatal	*Initial total*: 6822 *Mothers*: 6822 *Babies*: 6846 *Partners*:4404	To study outcomes and developmental trajectories to identify the main causal pathways across multiple levels of influence (political, social, cultural, intergenerational, familial and individual).	*Waves & Ages*:– 7; Antenatal, 6, 35 w, 9,12,16,23,24, 31, 4 y; then ongoing to 21 y *Retention rate*: 99% at 9 m	[36–38]
Peri/postnatal Epigenetic Study	Longitudinal Twin birth cohort	*Location*: Melbourne, Victoria, Australia. *Year & status*: 2007; Ongoing	*Period*: 32 months. *Age of*: Antenatal	*Initial total*: 287 *Mothers*: 250 *Babies*: 500 *Partners* -	To understand how adverse intrauterine environment predisposes individual to complex disease in later life.	*Waves & Ages*:*–* 5; 3 antenatal, birth, 18 m *Retention rate*: 97% 18 m	[39, 40]
Port Pirie Cohort Study–Birth to Now study	Prospective birth cohort	*Location*: Port Pirie, South Australia, Australia. *Year & status*: 1979; Ongoing	*Period*: 36 months. *Age of*: Antenatal	*Initial total*: 831 *Mothers*: 723 *Babies*: 723 *Partners* -	*Original–*To study the effects of lead exposure in utero and during childhood on later behaviour and development. *Current–*Long term follow-up of cohort with a focus on health, well-being, personality, emotions, behaviour and life experiences.	*Waves & Ages*:– 13; 2 antenatal, birth, 6,15 m, 2,3,4,5,6,7, 12, 28 y *Retention rate*: 47% at 28 y	[41–45]
Splash	Prospective birth cohort	*Location*: Barwon, Victoria, Australia. *Year & status*: 2012; Emerging	*Period*: N/A. *Age of*: Antenatal	*Initial total*: N/A *Mothers*: Aim for 500 *Babies*: Aim for 500 *Partners*: N/A	To study the impact of environmental, social and family level influences in child oral health and obesity risk factors.	*Waves & Ages*:–Antenatal, 6, 12,24,36,48 m planned *Retention rate*: N/A	[46]
The Mater-University of Queensland Study of Pregnancy	Prospective birth cohort	*Location*: Brisbane, Queensland, Australia. *Year & status*: 1981; Ongoing	*Period*: 36 months. *Age of*: Antenatal	*Initial total*:8458 *Mothers*: 7631 *Babies*: 7223 *Partners*:522	*Original*–To study the impact of social, obstetric factors and psychological factor on pregnancy outcomes. *Current*—To study health, growth, development, learning and behaviour of children and young adult.	*Waves & Ages*:– 7; antenatal; 4d, 6m, 5,14,21 y 30 y underway. *Retention rate*: 53% at 21y	[47–49]
The NZ Asthma and Allergy Cohort Study	Prospective birth cohort	*Location*: Christchurch and Wellington, New Zealand. *Year & status*: 1997; Ongoing	*Period*: 36 months. *Age of*: Antenatal	*Initial total*:1105 *Mothers*: 1095 *Babies* 1105 *Partners*: N/A	To study the environmental and genetic factors associated with the development of atopy, allergic disease and asthma.	*Waves & Ages*:– 8; Birth, 3,15,24,36,48,60 m, 6.5 y. *Retention rate*: 91% at 15 m	[50, 51]
The Raine Study	Randomised Controlled Trial, ongoing cohort followed	*Location*: Perth, Western Australia, Australia. *Year & status*: 1989; Ongoing	*Period*: 30 months. *Age of*: Antenatal	*Initial total*: 2900 *Mothers*: 2900 *Babies* 2868 *Partners*: 2804	*Original*—To test the hypothesis that complications of pregnancy might be prevented by frequent ultrasound, and to develop a long-term cohort to study the effects of early life events on later health. *Current*—To understand how events during pregnancy, as well as childhood and adolescence affect later health and development.	*Waves & Ages*:– 14; 2 antenatal, 2 d,1,2,3,5,8,10,14,17, 18,21,23 y *Retention rate*: 82% at 14 y	[52–56]
Triple B Study: Bumps, Babies and Beyond	Prospective birth cohort	*Location*: Sydney, New South Wales and Perth, Western Australia, Australia. *Year & status*: 2009; Ongoing	*Period*: 36 months. *Age of*: Antenatal	*Initial total*:1619 *Mothers*: 1619 *Babies* 1599 *Partners*: 852	To study the effects of substance use in pregnant women and their partners during the prenatal period on infant development and family functioning.	*Waves & Ages*:– 4; Antenatal, 8 w, 12 m, 3 y *Retention rate*: N/A	[57]
Watch Study	Prospective longitudinal cohort	*Location*: Newcastle, New South Wales, Australia. *Year & status*: 2006; Ongoing	*Period*: 18 months. *Age of*: Antenatal	*Initial total*:180 *Mothers*: 180 *Babies* 182 *Partners*: 82	To study whether maternal nutritional and hormonal factors are important predictors of offspring outcomes such as growth, body composition and childhood cognition.	*Waves & Ages*:– 11; 19,24,30,36 w antenatal; 3,6,9,12 m, 2,3,4, y *Retention rate*: 74% at 2 y	[58]
Aboriginal Birth Cohort	Prospective longitudinal birth cohort	*Location*: Darwin, Northern Territory, Australia. *Year & status*: 1987; Ongoing	*Period*: 38 months. *Age of*: Birth	*Initial total*^a^: 686 *Mothers*: 686 *Babies* 686 (includes subset) *Partners*: N/A	*Original*–To study the effects of foetal growth restriction on subsequent growth and development of chronic diseases in adulthood. *Current*–Effect of early life factors, birth and childhood, on later physical and mental health.	*Waves & Ages*: 4 waves–Birth, 11.4 y, 18.4 y, 25 y currently underway. *Retention rate*: 68% at 18.4 y	[59, 60]
Adelaide Nutrition Study	Prospective birth cohort	*Location*: Adelaide, South Australia, Australia. *Year & status*: 1975; Completed	*Period*: 8 months. *Age of*: Birth	*Initial total*: 500 *Mothers*: 267 *Babies* 200, 123 additional children at 11 y. *Partners* 219	To examine cardiovascular risk factors from birth.	*Waves & Ages*: 13 waves–Birth, 3,6,12 m, 2, 4,6,8,11,13,15, 17, 20 y. *Retention rate*: 50% at 20 y	[61–64]
Adelaide Respiratory Cohort	Prospective birth cohort	*Location*: Adelaide, South Australia, Australia. *Year & status*: 1987; Completed	*Period*: 12 months. *Age of*: Birth	*Initial total*: 1981 *Mothers*: 836 *Babies* 836 *Partners*: 573	To study the origin of respiratory diseases in children.	*Waves & Ages*: 6 waves–Birth, 5,12,18,24 m, 9 y. *Retention rate*: 83%	[65, 66]
Christchurch Health and Development Study	Prospective birth cohort	*Location*: Christchurch, New Zealand. Ye*ar & status*: 1977; Ongoing	*Period*: 6 months. *Age of*: Birth	*Initial total*:1310 *Mothers*: 1251 *Babies* 1265 *Partners*: N/A	To study the health, education and life progress of children born in Christchurch across infancy, childhood, adolescence and adulthood.	*Waves & Ages*: 22 waves–Birth, 4 m,1,2,3,4,5,6,7,8,9, 10,11,12,13,14,15,16,18,21, 25, 30, 35 y. *Retention rate*: 80% at 30 y	[67, 68]
Dunedin Multidisciplinary Health and Development Study	Prospective, longitudinal birth cohort	*Location*: Dunedin, New Zealand. *Year & status*: 1972; Ongoing	*Period*: 12 months. *Age of*: Birth	*Initial total*:1661 *Mothers*: 1037 *Babies* 1037 *Partners*: 753	*Original*: To study the health and development across the first 3 years of life. This initial aim was expanded to include health and development across childhood, adolescence and adulthood.	*Waves & Ages*: 13 waves–Birth, 3 5,7,9,11,13,15,18,21, 26, 32, 38 y. Planned 44, 50 y. *Retention rate*: 96% at 32 y	[67, 69–72]
Gudaga Study	Prospective, longitudinal, birth cohort	*Location*: Campbelltown, New South Wales, Australia. *Year & status*: 2005; Ongoing	*Period*: 18 months. *Age of*: Birth	*Initial total*: 2108 (surveyed) *Mothers*: 155 *Babies* 155 *Partners*:	To study the health, development and service usage of Aboriginal infants and their mothers.	*Waves & Ages*:– *6;* Birth, 2.5 w, 6 m, 1,3,5 y. *Retention rate*: 86% at 12 m	[73, 74]
Nepean Study/ Nepean Kids Growing Up Study	Birth cohort	*Location*: Penrith, New South Wales, Australia. *Year & status*: 1989; Completed	*Period*: 9 months. *Age of*: Birth	*Initial total*: 2508 *Mothers*: 2314 *Babies* 2314 *Partners*: 293	*Original*–to investigate the effects of birth size, body size and genes on blood pressure and bone density. *Current*—to explore foetal and mid-childhood influences, including family environment, on body composition, including bone and metabolic risk.	*Waves & Ages*:– 5; Birth, 6 m, 7.5, 12.5, 15 y. *Retention rate*: Not stated	[75–79]
Pacific Islander Families	Prospective, longitudinal, birth cohort	*Location*: Auckland, North Island, New Zealand. *Year & status*: 2000; Ongoing	*Period*:9 months. *Age of*: Birth	*Initial total*: 1590 *Mothers*: 1376 *Babies* 1398 *Partners*: 825	To study the factors associated with child health and development outcomes and family functioning of Pacific Islander families.	*Waves & Ages*:– 6; 6 w, 1,2,4,6, 9 y. 11 y planned. *Retention rate*:77% at 2 y	[80, 81]
Tasmanian Infant Health Study	A scoring system identified children at higher risk of SIDS for possible participation in TIHS	*Location*: Tasmania, Australia. *Year & status*: 1998; Ongoing	*Period*: 24 months. *Age of*: Birth	*Initial total*: 1500^a^ *Mothers*: 1433 *Babies* 1435 *Partners*: N/A	*Original*: To investigate the cause of Sudden Infant Death Syndrome. *Current*—To study links between early life exposures and later disease.	*Waves & Ages*:– 5; Birth, 5, 10 w, 7.5, 16 y. *Retention rate*: 415 at 16 y	[55, 82–86]
Brunswick Family Study	Longitudinal, birth Cohort	*Location*: Brunswick, Victoria, Australia. *Year & status*: 1978; Completed	*Period*: 6 months. *Age of*: 6 weeks	*Initial total*: 304 *Mothers*: 304 *Babies* 272 *Partners*: N/A	To study the prevalence and patterns of illness and behavioural disturbances in infants.	*Waves & Ages*: - 5; 6,27,44 w, 4, 11 y. *Retention rate*: Unknown	[87]
Plunket National Child Health Study	Prospective longitudinal cohort	*Location*: New Zealand, national. *Year & status*: 1990; completed	*Period*: 12 months. *Age of*: 6 weeks	*Initial total*: 4285 *Mothers*: 4285 *Babies* 4285 *Partners*: Unknown	To study community child health, maternal health behaviour, parental attitudes, child safety, nutrition and morbidity.	*Waves & Ages*:– 9; 6 w, 3,6,9,12, 18m, 2,3,4 y. *Retention rate*: Unknown	[88]
VicGen	Prospective birth cohort	*Location*: Metropolitan and regional and rural Victoria, Australia. *Year & status*: 2010; Emerging	*Period*: 72 months. *Age of*: 4 weeks	*Initial total*: 466 *Mothers*: 466 *Babies* 466 *Partners*: N/A	To study the natural history of early childhood caries including the prevalence of the disease, risk and protective factors	*Waves & Ages*:– 1,6,12,18, 36,48, 60, 72m. *Retention rate*: N/A	[89]
Total				*Initial total*: 44,071 ^b^ *Mothers*: 38,600 *Babies* 38,390 *Partners*:14,206			

^a^ Data available on 8729 mothers and 10569 babies, follow up study with 1500

^b^ Initial total is generally the number of mothers whose data was collected and from that point onwards, some or all were invited to participate in a follow-up study.

### Conceptual Framework and Aims of Studies

Many of the studies had a DOHaD focus. Some studies specify chronic diseases or factors related to disease causation i.e. blood pressure or asthma, while other studies are more broad in examining the effects of factors in utero and early childhood on later health outcomes. Several emerging studies have a targeted focus including early childhood dental caries (decay) or asthma and allergies. Yet the data collected are still likely to be applicable in examining other health issues including obesity, which as de Silva et al. [46] argue share many common risk factors.

Of the 23 studies included, 16 articulate an explicit or broadly defined conceptual framework that informs the research methodology. Five studies document applying a DOHaD approach [39, 52, 58, 59, 75], four studies have a broad focus on human health and development [47, 69, 90, 91], whilst a further three studies explicitly state using an ecological framework to study human development [31, 36, 89]. Two studies examine the origins of respiratory diseases, atopy and/or allergic diseases [50, 66]. Of the remaining two studies, one used an integrated chronic disease prevention framework [46] and the other study used a social reciprocal framework of community engagement, respect and equality [92]. Seven studies do not identify a conceptual framework.

Of the 23 included studies, 17 were located in Australia and 6 in New Zealand. Three of the included studies were focused on Indigenous populations; two are located in Australia Australian Aboriginal Birth Cohort Study [60] and the Gudaga Study [73] and one in New Zealand The Pacific Islands Family Study [80].

### Status of Studies

Of the included studies, five studies commenced in the 1970’s, five in the 1980’s, four in the 1990’s, seven in the 2000's and two commenced after 2009. The longest running study is the Dunedin Multidisciplinary Health and Development Study, which has been running for over 41 years since 1972. Of the 23 studies, 22% (n = 5) have been completed, 70% (n = 16) are ongoing, and 9% (n = 2) are emerging studies that have commenced data collection, but currently have only protocol papers.

### Design of Studies

Predominately the studies are of prospective longitudinal birth cohort design (83%, n = 19). Of the remaining four studies, one is a twin prospective longitudinal birth cohort, one study commenced as a randomized controlled trial (RCT) and one identified children at higher risk of SIDS for possible participation. One study conducted interval sample recruiting, where participants were recruited for 4 months of the year, for a total of 5 years.

### Study Size

The initial sample study sizes ranged from 180 to 8458 participants, with a median of 1590 at intake. The number of recruited liveborn babies ranged from 155 to 7223, with a median of 1105. Eleven of the 23 studies included the participation of fathers, with a median of 82 fathers participating as shown in Table 2. Two-thirds of studies recruited more than 1000 participants.

**Table 2:** Number of participants in included studies (ongoing or completed).

	Number of mothers	Number of babies	Number of partners	Total
Median	1095	1105	82	753
Total Participants	38600	38390	14206	91196

Note: N = 21. Two studies had not completed recruitment therefore are not included.

### Commencement of Data Collection

Eleven (48%) of the studies commenced data collection in the antenatal period and nine studies commenced at birth (39%) or shortly post-partum. A further three studies commenced data collection in the first 6 weeks of life. The 21 established studies each had a range of data collection waves, between 4 and 22 follow-up occasions (mean 8.4, SD 4.5, median 7).

### Data Collected

The diverse range of data collected in these birth cohort studies is shown in Table 3. All studies included a physical assessment at some point. Anthropometric measures were commonly collected in birth cohort studies, with many studies collecting this data at every wave, although a small number of studies only collected birth measurements. Health, mental assessments and nutrition assessments were are also regularly collected. Biological assessments were common and samples obtained included blood, saliva, urine, cord blood, placental tissue, and hair, nail, and teeth samples (Table 4).

**Table 3:** Types of Data Collected From Included Studies.

Data collected	Total no. of studies	Percentage of studies	Child/ Adolescent	Mother	Father	Primary Caregiver
Anthropometric	17	74%	17	11	6	0
Biological^a^	16	70%	17	8	3	2
Genetic	9	39%	9	3	2	1
Medical records	18	78%	17	15	1	1
Spatial resolution data	2	9%	2	1	1	0
**Assessments Performed**						
Physical Environment	4	17%	4	3	0	1
Cognitive	7	30%	7	2	1	0
Nutrition	17	74%	16	12	2	3
Infant Development	17	74%	17	0	0	0
Mental Health	16	70%	11	9	6	3
Physical Health	23	100%	23	18	7	3

^a^Biological–blood, saliva, urine, cord blood, placental tissue, and hair, nail and teeth samples.

**Table 4:** Biological Samples Collected in Included Studies.

Sample Type	Studies
Buccal swab or saliva sample	Peri/postnatal Epigenetic Study, Nepean Hospital, Watch Study, Bumps, Babies and Beyond Study, VicGen
Child/Adolescent blood	Growing up in New Zealand, Watch Study, Port Pirie Cohort Study, Dunedin Multidisciplinary Health and Development Study, Australian Aboriginal Birth Cohort Study, Raine Study, NZ Asthma and Allergy Cohort Study, Adelaide Nutrition, The Mater-University of Queensland Study of Pregnancy Study, Christchurch Health and Development Study, Generation 1, Nepean Study, Tasmanian Infant Health Study
Cord blood	The Raine Study, Peri/postnatal Epigenetic Study, Tasmania infant health study, Environments for Healthy Living Griffith Birth Cohort Study, New Zealand Asthma and Allergy Cohort Study, Watch Study
Faecal	No studies identified
Hair	NZ Asthma and Allergy Cohort Study
Maternal blood	Growing up in New Zealand, Watch Study, Raine Study, Port Pirie Cohort Study, Peri/postnatal Epigenetic Study, Tasmanian Infant Health Study
Toe Nails	Splash
Paternal blood	Growing up in New Zealand, The Raine Study,
Placental tissue	Peri/postnatal Epigenetic Study
Primary Caregiver blood	Environments for Healthy Living Griffith Birth Cohort Study, VicGen
Teeth	Port Pirie Cohort Study, Christchurch Health and Development Study
Urine	Australian Aboriginal Birth Cohort Study, Tasmanian Infant Health Study

Genetic research has been undertaken by many of the studies. Based on the 736 full-text articles included in this review that span the 23 studies, genetic research has focused on intelligence [93], obesity and adiposity [85, 94, 95], mental and behavioural disorders [96–100], epigenetic changes [39], dyslexia [101] and eye health [102] (Table 5).

**Table 5:** Genetic Areas Analysed in Included Studies.

Genetic Areas	Included studies
Intelligence	Copy number variation and intelligence [93]
Obesity and adiposity	Genome-wide association studies of obesity related phenotypes [94]; APOE genotypes and adiposity [85]; 17 genetic loci (FTO, MC4R, TMEM18, GNPDA2, KCTD15, NEGR1, BDNF, ETV5, SEC16B, LYPLAL1, TFAP2B, MTCH2, BCDIN3D, NRXN3, SH2B1, MRSA) associated with childhood obesity [95]
Mental and behavioural disorders and problems	Catechol O methyltransferase gene (COMT) and aggression and attention problems[96], MAOA genotype and antisocial behaviours [97], serotonin transporter genes (5-HTTLPR) and mental disorders [98], monoamine oxidases A and B (MAOA, MAOB), dopamine beta hydroxylase (DBH), phenylalanine hydroxylase (PAH), tyrosine hydroxylase (TH) & the dopamine receptor 5 (DRD5)—coupled with SNP analysis at the COMT, DRD1, DRD2 & DRD3 loci and behavioural disorders [99], genetic association analysis and anxiety and depression [100]
Other	Gene expression and epigenetic changes [39]; Dyslexia candidate genes MRPL19/C2ORF3, KIAA0319, DCDC2 and DYX1C1 and literacy [101];To identify early features of Fuchs endothelial dystrophy (FED) in carriers of the rs613872(G) transcription factor 4 gene (TCF4) [102]

Some studies have accessed data from other sources to link to their primary data collection. In New Zealand, the Growing up in New Zealand study has linked with the Ministry of Health, the National Minimum Data Set and the National Immunization Register [36]. The Christchurch Health and Development Study has accessed police record data from when the participants were age of 14, as well as hospital records across childhood [90]. The Dunedin Multidisciplinary Health and Development Study are similarly using police record data, health data including that of general practitioners, emergency service and hospital records. The investigators are using traffic accident reports and police traffic records for studies of injury, health and drink driving. Credit and government benefits are being accessed for studies of work and finances [72]. In Australia, the Raine Study has linked education [103], child protection and justice data sets to their data [52]. The Mater-University of Queensland Study of Pregnancy has used child protection and juvenile justice data to explore whether a history of family social disadvantage or child abuse and neglect explain the overrepresentation of Indigenous Australian young people in youth detention [104]. Newer Australian studies are also accessing linked data, with the Environments for Healthy Living Griffith Birth Cohort Study accessing Medicare every five years [31], while the Gudaga study used Admitted Patient and Medicare data [73].

### Pooled Data

The systematic review revealed 14 articles that were based on pooled data from one or more birth cohort studies. The studies that pooled data were generally from the longer running cohorts, particularly the Dunedin Multidisciplinary Health and Development Study, the Christchurch Health and Development Study and the Raine Study. The majority of the articles reported on pooled data with international studies, with only one based on Australian studies [105]. Nine articles pooled data from two different birth cohort studies [106–112], 2 articles reported on data from 3 cohort studies [113, 114], one article reported on data from four birth cohort studies [115] and one article reported on data from 5 birth cohort studies [116]. One article pooled data from 23 studies across 4 countries [117].

Topics covered in the pooled data articles extended from influences on anthropometric measurements to include genetic and environmental contributions [117], genetic variants associated with foetal growth and birth weight [108], and height and weight comparisons between children from two countries [109]. Influences on cognitive outcomes were also explored through pooled data sets including the relationship between birth weight and IQ [107], sex differences in developmental reading ability [115] and the neurobehavioral effects of lead [116]. The effects of mental health and neurodevelopmental functioning on behaviour, peer affiliation and intelligence have been thoroughly investigated [111, 113, 118].

### Open Access Data

Many of the Australian and New Zealand ongoing cohort studies have mechanisms in place for collaborative access to the data, but no cohort data, to our knowledge, are available open access. Certainly none of the included studies have been developed to facilitate open access to all data collected. Sudlow et al. argue this open access to the global research community is crucial in maximising the impact of birth cohort studies on the policy and scientific knowledge [119].

## Discussion

This systematic review of Australian and New Zealand birth cohort studies identified 23 studies. Like many things, cohort studies improve with age—the oldest trial in this review commenced in 1972 [120] and has generated 1150 publications and reports. Many of these articles have become citation classics (e.g. one article has over 5000 citations) [121]. This review revealed several themes that should be considered for future research particularly during the development and establishment phases such studies.

### Theme 1: Aim Changes

It is evident that over time that many of these studies have contributed to awareness, practice and policy changes that make their original objectives unnecessary or unproductive. Of the 23 birth cohort studies, six have expanded their original aims into a broader and more longitudinal focus. For example, the Tasmanian infant health study, that was designed to investigate the causes of Sudden Infant Death Syndrome, successfully contributed to changing policy and practice reducing infant mortality, but subsequently expanded to investigate the links between early life exposures and later disease development. Another example is the Port Pirie Cohort Study which began with a focus on lead exposure and development in-utero and throughout childhood, and now has a broader focus on other factors that influence health and well-being across the lifespan.

The substantial resources and investment required to establish and maintain a birth cohort study [122], as well as the breadth of prospective data collected, means that it is advantageous and may be more economical to adapt existing narrower studies into ongoing longitudinal studies of a broader nature. Indeed several new and emerging cohorts are developed in a way that supports the extension beyond the initial aims [39]. This means, therefore, that there is an imperative to ensure baseline measures have significant breadth to encompass a change of aim from the initial focus to reflect long-term pathways and outcomes, and also to facilitate the sharing of data between cohorts. For future birth cohorts, maternal and paternal data collection would be an additional priority to understand the relationships between genes, lifestyle and environment, during the peri-conceptional, pregnancy and childbirth periods. The interest in epigenetics continues to grow as this offers the potential to elucidate some of the complex mechanisms through which genetic and environmental factors contribute to health and development [21].

### Theme 2: Geographic Distribution

There is continued interest in undertaking studies in specific geographic regions (microclimates), such as studies in Dunedin, Christchurch, and Port Pirie, to understand regional influences on the health and structure of the population, and how that compares with broader population data. The use of microclimates may offer a useful avenue to address the issue of funding and resources for future birth cohort studies, as large geographically disperse samples are often cost prohibitive to recruit and retain [36]. A microclimate offers advantages of being able to target publicity in an ongoing way and develop a local identity for the study [36, 70]. Furthermore, local area institutional facilities can be used for assessments in a consistent way [123]. Undertaking a birth cohort study in a discreet region supports the translation of evidence to service provision in that area an aim of both current [73] and emerging region-specific birth cohort studies (The Illawarra Born cross-generation health study) [124].

### Theme 3: Participants

Most studies have focused much of their research efforts on the child, with some maternal data collection, typically in the antenatal and post-partum period. Several long standing studies have continued to collect maternal data, including the Mater-University of Queensland Study of Pregnancy and the Raine study. These studies have contributed to further understanding the mothering, maternal health and midlife experiences of women as well as the intergenerational transmission of depression, drinking and smoking [125].

Collection of data from fathers is an emerging area, but currently only half (48%) of studies have collected data on at least one occasion from fathers. Growing Up in New Zealand collected data from fathers before birth [37], the authors argue this prospective involvement is unique in the New Zealand context. Recruiting and retaining fathers continues to be a challenge for studies [126]. One of the changes evident in the more recent birth cohorts is the collection of parent data from the ‘primary caregiver.’ Splash, VicGeneration, Environments for Health Living and Generation 1 studies all use the primary caregiver (female or male) as the parental informant post birth [31, 33, 46, 89]. The collection of data from primary caregivers will require careful definition in data sets and stratification to delineate the relationships that offer environmental and genetic influences on the index child.

The Mater-University of Queensland Study of Pregnancy also collected data on 520 siblings born in the first three years of the study, enabling the study of family characteristics on child outcomes [47]. Other informants in birth cohort studies have included teachers: the Christchurch Health and Development Study [90], the Mater-University of Queensland Study of Pregnancy [127] and the Dunedin Multidisciplinary Health and Development Study [128]. The Pacific Islander Families Study also plans to interview teachers [91].

Several cohorts have had extended periods of no communication with participants (up to 15 years) before attempts were made to re-establish the cohort for further data waves. The Port Pirie Cohort Study undertook extensive data collection in the first seven years of children’s lives, with a sub sample being followed from 12 years of age. There was then no contact until the age of 27 [44]. The Aboriginal Birth Cohort study commenced data collection at birth and there was eleven years before the next data wave, and then the next follow-up was a further seven years later [59]. The Port Pirie study firstly reviewed the National Death Index to identify deceased participants and the remaining were sourced through the Australian Electoral Commission and the White Pages as well as contact through their parents [44]. They achieved a retention rate of 47 per cent of participants and contact details were unable to be located for 22% [44]. The Aboriginal Birth Cohort study has articulated how they addressed the logistical, cultural and linguistic challenges they faced in retaining 83 per cent of their participants at the 18 year follow-up across urban and rural communities [129]. The Aboriginal Birth Cohort study used the same approaches as the Port Pirie study, but in addition the study team widely engaged with community members and community groups to locate participants over a twelve month period [130].

### Theme 4: Intergenerational Studies

There is increasing interest and acknowledgment of the value in collecting detailed data on parents and grandparents, not just the index child [126]. The Dunedin Multidisciplinary Health and Development Study (n = 1037) has conducted a number of sub studies including following on with the original cohort members’ children (The Next Generation Study) as well as investigating parents of the Dunedin cohort (Family Health Study) [131]. Future studies would be well placed to investigate intergenerational influences commencing in the peri conception period and in utero [132].

### Theme 5: Data Collection

A key challenge for birth cohort study investigators is the resources required for regular data collection [50]. The majority of studies collect data in person with participants. As an example, the Dunedin Multidisciplinary Health and Development Study bring in participants at least every five years, within 2 months of their birthday for up to a full day [70]. Other studies have addressed the resources issue by collecting supplementary data. For example, Growing up in New Zealand supplemented big waves of data with brief (5 minute) computer operated phone interview data collections 6 and 35 weeks, and 16 and 23 months post-partum [36].

Many of the included birth cohort studies utilise data linkage opportunities to reduce participant burden and maximize use of their resources. Data sets utilised include health, prescription, disease and death registries, education, child protection and justice data sets. McKnight et al [52] argue that linking birth cohorts to total population data sets is an effective and inexpensive way to increase information on the cohort, particularly those lost to follow up. Furthermore, this data can increase the understanding of how representative the cohort is in terms of the general population [52].

### Theme 6: Pooling of Data

Pooling data or undertaking cross cohort comparisons supports an understanding of which aspects of children’s development are unique to specific contexts and which are universal [132]. Advantages of pooling data include replication of findings [106, 118], increased statistical power and confidence [117, 133], appreciation of gender, cultural and geographical differences on results [106], and the capacity to answer research controversies or conflicting findings [115]. Challenges related to pooling data include differences in procedures and sampling [109], type of assessment and measurements [108], recording of data and the limitations with statistical fitting of models [133]. Some issues can be addressed by greater convergence of assessment and procedures [106], however there are also examples of where differences in samples and assessments can strengthen research findings [111]. The accessibility of study protocols and public release of sampling and measurement details can further support combining cohorts or conducting cross cohort comparisons [23]. Additionally, networking among birth cohort study investigators through forums such as the ARACY longitudinal studies network supports emerging studies to consider possibilities for collaborating and pooling data. A system that support data sharing and information about birth cohort studies has been established internationally through the European Birth Cohort Network (www.birthcohorts.net) [126], and several Australian and New Zealand studies are included. Nonetheless, there are compelling reasons for more to be done in future in relation to adopting open access data sharing models where participants provide broad consent for future research inquiry.

### Theme 7: Contributions to Policy Development

Vimpani, Patton and Hayes [134] argue for the importance of a research informed policy agenda in the fields of developmental health and well-being. Long running birth cohorts have been in a position to significantly influence policy through their research in a variety of fields including health, social and educational development and safety.

The Christchurch Health and Development Study has made important contributions in a number of policy development areas including the utilisation of childhood services by disadvantaged families, the importance of early intervention to avoid multiple problems as well as new understanding of the long term outcomes of cannabis use, and also how conduct problems in younger years affect later development. The principal investigators have also sought to use their findings to influence public health issues such as swimming pool safety, effects of passive smoking on children, impact of sub-clinical lead levels on child development and suicide and mental health awareness [68]. The Port Pirie Cohort Study in addition to the Christchurch Health and Development Study, have similarly sought to highlight sub-clinical levels of lead exposures on later cognitive outcomes and emotional and behavioural problems [135–137] and have resulted in the design and conduct of retrospective studies that then have been used to influence public health policy. The Dunedin Multi-disciplinary Health and Development Study has also contributed to improvements in policy and practices in child and adolescent development. The principal researchers argue for a balance between scholarly research and application, by producing a range of reports for government departments and other agencies [69].

It is also important to recognise that many of the birth cohorts have had an immediate impact on service delivery. The Gudaga Study [73] collaborated with health and child welfare stakeholders to improve policy and practices and service provision to Aboriginal children and their families. The Raine study was initially conceived as a randomized controlled trial to of frequent prenatal ultrasound to prevent complications of pregnancy [53], but subsequently this was developed into a longitudinal birth cohort. The early findings of this study suggested frequent ultrasounds should only be used when clinically important.

There is significant yield from this effort both in terms of scientific discovery and social policy impact. Fergusson and Horwood [90] argue longitudinal birth cohorts meet the interests of multiple endusers including the research community, policy makers and clinicians, by providing cost effective, methodologically robust and theoretically grounded research. Furthermore, longitudinal studies offer the flexibility to address emerging research questions [90], as well as informing current and future public policy [132]. As Lawlor et al [126] argue, new birth cohort studies are a resource for the present as well as the future.

There were study limitations. The search for Australian and New Zealand birth cohorts was systematic and extensive, and limitations relate to the reporting on study data and accuracy. Campbell and Rudan [20] in their systematic review of birth cohort studies in the sub-Saharan region of Africa, highlight the challenges in reviewing the long-standing birth cohorts to capture the most recent description of studies and to include all of the data collected. It is possible that this review may have missed some data capturing, particularly unreported data or data reported in publications that were not identified through the systematic review. Furthermore some of the data collected from the analysis of the 23 studies was extracted from study protocols. Not all included studies have a published protocol and there is also the possibility that some data may not have been collected as planned, or extra data may have been added. Some of the larger studies have published between 400 and 690 publications [68, 72] and not all publications were reviewed, thereby some data assessments undertaken by the included studies may have been missed. Of the 19 experts who were contacted from each of the 23 studies, ten did not respond and were therefore unable to assist us in the identification of additional studies. This limitation was addressed by contacting another principal investigator when an automatic out of office response was received from an expert with that study.

The last decade has seen a growth in the number of birth cohort studies, with a least one commencing per year. Given the continual changes in society through technological and medical advancements, climate change, globalisation and social connectedness, continued research is required to understand how families and the developing child respond to these changes. In Australia significant new policy initiatives may further alter the parenting experience, such as a proposed disability insurance scheme and extensions to paid parental leave. In New Zealand, welfare reforms related to improving outcomes for vulnerable children, and the findings of the recent inquiry into child health and preventing child abuse, recommended significant policy emphasis on the period of pre-conception to three years.

Cohort studies do also have some limitations, they are hypothesis generating, and trend trackers but they do not test hypotheses or provide causal information. Notwithstanding this, birth cohort studies offer a significant opportunity to understand how these social policy and public health changes affect the whole family unit and to inform future public policy. Primarily most studies place their focus on the newborn child and their journey through infancy, childhood, adolescence and adulthood, but several studies have taken on a broader intergenerational focus. This focus allows exciting new areas of research inquiry in understanding human development through the framework of the family unit whose environment, genetic and behaviours all leave a lasting legacy on the child.

Despite the demonstrated value of birth cohorts in contributing to scientific knowledge and policy and practice, funding remains a significant challenge for new and ongoing birth cohort studies. Indeed some of the emerging studies only outline data collection and follow-ups for the first few years. Funding cycles of grants typically encourage tightly focused studies of limited duration. This review provides evidence of a growing number of emerging birth cohort studies more singular in focus. It appears to be difficult to get a broad based birth cohort study funded as granting bodies can be reluctant to take on a study that may take decades of commitment [106]. Many long running studies have articulated the difficulties that insufficient funding has had on their capacity to collect and manage data [47, 52]. Several established studies in Australia and New Zealand have mitigated this issue by successfully applying for funding for the initial few years and then again as the cohort of children or young people reach key transitions e.g. commencing school or parenthood. Undertaking research in discrete microclimates may be another way of managing limited resources in the costly data collection and participant retention phases.

## Conclusions

Birth cohort studies have significantly contributed to the understanding of human development. There is, however, a great deal more to learn about child development and its impact on future outcomes. Exciting new advances in the fields of genetics and epigenetics offer researchers opportunities to expand the current knowledge on the interplay between genes and environment on outcomes, in a cost effective way. Opportunities to collaborate and pool data across national and international borders and providing open access to data mean that birth cohort studies can advance scientific knowledge and practice from a local level up to global challenges.

## Supporting Information

S1 PRISMA Checklist PRISMA 2009 Checklist. (DOC)
